# Editorial: Rising stars in PET and SPECT: 2022

**DOI:** 10.3389/fnume.2023.1326549

**Published:** 2023-11-10

**Authors:** David Izquierdo-Garcia, Seung Kwan Kang

**Affiliations:** ^1^ Athinoula A. Martinos Center for Biomedical Imaging, Department of Radiology, Massachusetts General Hospital, Boston, MA, United States; ^2^ Harvard Medical School, Boston, MA, United States; ^3^ Harvard–MIT Division of Health Sciences and Technology, Cambridge, MA, United States; ^4^ Bioengineering Department, Universidad Carlos III de Madrid, Madrid, Spain; ^5^ Institute of Radiation Medicine, Medical Research Center, Seoul National University, Seoul, Republic of Korea; ^6^ Brightonix Imaging Inc., Seoul, Republic of Korea

**Keywords:** PET, SPECT, cardiac imaging, radiotheranostics, deep learning, attenuation correction (AC), reconstruction-free PET

**Editorial on the Research Topic** Rising Stars in PET and SPECT: 2022

On this new feature of Frontiers in Nuclear Medicine, we are delighted to present the Research Topic *Rising Stars in PET and SPECT: 2022*. We would like first to thank all the contributors for their great quality on their submissions, which have undoubtedly enhanced the depth and quality of this compilation. We want to highlight that all these manuscripts have been originated from young talented researchers that are already becoming the stars in the PET and SPECT fields that will shine us the light to guide our future and our path to excellence. It has been a terrific Research Topic with high-quality manuscripts, and for us, as Editors, it has been an immense pleasure to be part of it.

In this Research Topic you will find an interesting variety of topics, from reviews on cardiac PET/MRI for sarcoidosis applications and preclinical antibody PET imaging for oncologic applications, to more technical studies such as the use of the background radiation of the Lutetium detectors on the TotalBody scanner or the use of Deep Learning applications for automatic delineation of tumor areas in pediatric gliomas, to a final position paper that challenges the future of reconstruction-free ultra-fast ToF PET scanners.

It is with great anticipation that we offer readers a glimpse into the captivating essence of these works, recognizing the profound contributions that each article brings to the broader discourse of PET and SPECT research. We invite our readers to embark on a journey of discovery through these pages, where the boundaries of current knowledge are constantly being pushed and redefined, promising a future enriched by innovation and progress. We hope the reader will forgive us for unveiling some of the really interesting details that these works have put together, but we believe is worth walking the reader briefly through them.

In the first place, the work by Munoz et al. touches one of the most challenging areas in cardiac imaging: the diagnosis of sarcoidosis. Sarcoidosis is a multisystemic disorders that mainly affects the lungs, but can affect other organs, including the heart. There is currently no gold-standard for diagnosing sarcoidosis, although PET and MRI, have shown promising results separately in detecting some of the features that allow an accurate diagnosis, but each has its limitations. The use of the integrated PET/MRI scanners has enabled the synergistic fusion of PET and MRI features to provide a better understanding of the underlying disease and hence to provide a more accurate diagnosis. The work of Munoz et al. an outstanding expert in cardiac PET/MRI analysis, especially in MR-based motion correction, is an excellent review that highlights the pros and cons of the different techniques and unveils some of the potential paths to further improve clinical diagnosis using PET/MRI.

Secondly, the work presented by Brown et al. lead by Dr. Pereira, one of the most talented young PET imaging researchers in the area of cancer biology and radiotheranostics, focuses on one the most promising areas within PET imaging: the use of antibody imaging to improve clinical decisions for cancer therapy. The authors describe the development and evaluation of various radiolabeled antibodies and antibody fragments that can specifically target Programmed cell death protein-1/ligand-1 (PD-1/PD-L1) in different tumor models. However, it's important to note that not all PD-L1-positive tumors respond to immunotherapy, and some patients with PD-L1-negative tumors have shown clinical benefit. This discrepancy is due to the inherent spatial and temporal heterogeneity of PD-L1 tumor expression, which is not accurately captured by biopsy-based diagnostic tests. In particular, the team highlights the first-in-human study of the clinically approved anti-PD-L1 antibody atezolizumab labeled with the positron emitter zirconium-89. They also discuss how PET imaging can reveal the spatial and temporal heterogeneity of PD-L1 expression, which has implications for patient selection and treatment monitoring. The article highlights the potential of molecular imaging of PD-L1 as a complementary tool for clinical oncologists and a powerful method to study cancer biology.

Third, Ladefoged et al. lead a novel work on the use of Deep Learning applications for the automatic delineation of tumors on pediatric gliomas. The use of Deep Learning (DL) has recently exploded for its applications in non-invasive imaging in the last years. One of the main areas of applications and interests in the community is the use of DL to facilitate or guide the automatic delineation of disease areas. In oncological applications, automatic tumor delineation has been a vast field of study. In this study, Dr. Ladefoged, a world expert in PET/MRI among others an international consortium that review and set the basis for improved brain PET imaging using PET/MRI scanners using accurate MR-based methods, presents their latest results on the application of artificial neural networks (ANN) for pediatric gliomas. The authors use an artificial neural network (ANN) that is pre-trained on a large cohort of adult patients with brain gliomas and then fine-tuned on pediatric patients. The ANN achieves high accuracy and concordance with manual delineation and provides clinically relevant metrics, moreover, performs well in longitudinal studies, showing consistent changes in the metrics over time. It has been well demonstrated that their method has the potential to serve as a complementary tool for clinical oncologists, as well as a powerful means to study cancer biology.

Fourth, the work of Omidvari et al. shows for the first time the potential of background radiation from the lutetium detectors in a TotalBody scanner. Dr. Omidvari is a young talented PET researcher who has been incredibly active in the field of TotalBody scanners, in our opinion one of the most amazing advances in the last decade in terms of PET technology. TotalBody scanners is a technology that has been made possible by a myriad of researchers at UC Davies, including but not limited to Drs. Badawi, Qi and Cherry, all of whom are co-authors on this incredible work. This novel research re-takes a long-forgotten idea of using the background radiation of the PET detectors as a potential source to provide a transmission scan to be used for attenuation correction, among others. The new technology from TotalBody scanners, mainly the vast number of the detectors and the incredible sensitivity provided by the scanners provides to this technology a second chance to proof its amazing capacity and its utility. The authors conducted a simulation study using Monte Carlo simulations of a 3D whole-body XCAT phantom in the uEXPLORER PET scanner. They focused on ultralow-dose PET scans that are now possible with these scanners and studied the effects of an increased acceptance angle, reduced scan durations, and Compton scattering on PET quantification. The authors compared different methods for reconstructing the attenuation maps from the lutetium background data, and evaluated their effects on PET quantification and image quality. The results showed that quantification and lesion contrast were minimally affected in both long axial field-of-view scanners and in a whole-body 20-min scan. A must-read paper for the lover so PET technical solutions.

Finally, the position paper by Schramm, a young and very talented scientist who has been working since the rise of combined PET/MRI scanners, challenges for the first time a unique concept and objective towards which many PET researchers have been turned into: the aim to obtain reconstruction-free PET images using ultra-fast time-of-flight (ToF) detectors. For the last years, there has been a lot of traction in the community, including several position papers in which the community is targeting the objective of attaining the limit of 10 ps time-resolution scanners to enable this reconstruction-free PET images. While the challenge is huge and opens the community to track and follow this path, Dr. Schramm, raises questions about the enormous technical challenges that are required to make this aim possible, and the long distance that current technology needs to travel in order to reach it. For example, the author argues that quantitative PET imaging still requires the calculation of line integrals to model photon attenuation, the estimation of scattered coincidences, and the suppression of noise, which are not feasible without iterative techniques or advanced modeling. A fascinating manuscript that will not leave anybody indifferent and will generate, without any question, a lot of attention.

The study by Munoz et al. explores the potential of hybrid PET-MR imaging for cardiac sarcoidosis. This approach combines the strengths of PET and MRI to provide a more comprehensive view of the disease. However, as you rightly pointed out, more research is needed to fully understand its potential and limitations. Brown et al.’s work on preclinical antibody-PET imaging of PD-L1 represents a significant step forward in cancer treatment. The use of antibody imaging to improve clinical decisions for cancer therapy is promising, but further research is needed to fully understand its potential and limitations. The use of deep learning methods for automatic detection and delineation of pediatric gliomas is another significant advancement presented by Ladefoged et al. While these methods can automate tumor delineation, further research could be done to optimize the ANN for different types of tumors or clinical settings. Omidvari et al.’s work on lutetium background radiation in total-body PET scanners presents a novel approach to PET attenuation correction. This study underscores the potential of using Lutetium background radiation for attenuation correction in total-body PET. However, it also highlights the need for further research to optimize scan parameters and implement appropriate correction methods to improve PET quantification accuracy. Lastly, Schramm’s position paper challenges the concept of achieving reconstruction-free PET images using ultra-fast ToF detectors. While this goal has gained significant traction in recent years, Schramm raises important questions about the substantial technical hurdles that must be overcome to realize this goal.

In summary, the articles presented in this Research Topic highlight the transformative potential of nuclear medicine to improve the diagnosis and treatment of disease. By harnessing the power of cutting-edge imaging techniques, machine learning algorithms, and innovative radiotracers, researchers are redefining the frontiers of medical imaging. However, as with any rapidly evolving field, there are hurdles to overcome. Persistent research efforts and collaborative endeavors will be instrumental in navigating these challenges and realizing the full potential of these groundbreaking approaches. We trust that readers will find the works presented in this Research Topic as compelling as we have. It is our hope that they derive as much pleasure from reading these articles as we have had in curating this Research Topic over the past months. We also encourage our readers to keep an eye on the future contributions of these promising young talents, as they are undoubtedly among the luminaries guiding our journey forward.

